# Epidemic size, trend and spatiotemporal mapping of SARS-CoV-2 using geographical information system in Alborz Province, Iran

**DOI:** 10.1186/s12879-021-06870-6

**Published:** 2021-11-25

**Authors:** Kourosh Kabir, Ali Taherinia, Davoud Ashourloo, Ahmad Khosravi, Hossien Karim, Hamid Salehi Shahrabi, Mojtaba Hedayat Yaghoobi, Alireza Soleimani, Zaynab Siami, Mohammad Noorisepehr, Ramin Tajbakhsh, Mohammad Reza Maghsoudi, Mehran Lak, Parham Mardi, Behnaz Nouri, Mohammad Mohammadzadeh, Mehdi Azimzadeh, Mahmood Bakhtiyari

**Affiliations:** 1 Department of Community Medicine, School of Medicine, Alborz University of Medical Sciences, Karaj, Iran; 2 Department of Emergency Medicine, Alborz University of Medical Sciences, Karaj, Iran; 3 Remote Sensing and GIS Research Center, Shahid Beheshti University, Tehran, Iran; 4 Department of Epidemiology, School of Public Health, Shahroud University of Medical Sciences, Shahroud, Iran; 5 Department of Cardiology, School of Medicine, Alborz University of Medical Sciences, Karaj, Iran; 6 Department of Infectious Diseases, School of Medicine, Alborz University of Medical Sciences, Karaj, Iran; 7 Department of Environmental Health, School of Health, Alborz University of Medical Sciences, Karaj, Iran; 8 Department of Internal Medicine, School of Medicine, Alborz University of Medical Sciences, Karaj, Iran; 9 Department of Infectious Diseases, School of Medicine, Shahid Beheshti University of Medical Sciences, Tehran, Iran; 10 Student Research Committee, School of Medicine, Alborz University of Medical Sciences, Karaj, Iran; 11 Department of Microbiology, School of Medicine, Alborz University of Medical Sciences, Karaj, Iran; 12 Information Technology Unit, Alborz University of Medical Sciences, Karaj, Iran; 13 Non-Communicable Research Diseases Center, Alborz University of Medical Sciences, Karaj, Iran

**Keywords:** COVID-19, Basic reproductive number, Case fatility rate, Geographic information system, Iran

## Abstract

**Background:**

The first confirmed cases of COVID-19 in Iran were reported in Qom city. Subsequently, the neighboring provinces and gradually all 31 provinces of Iran were involved. This study aimed to investigate the case fatility rate, basic reproductive number in different period of epidemic, projection of daily and cumulative incidence cases and also spatiotemporal mapping of SARS-CoV-2 in Alborz province, Iran.

**Methods:**

A confirmed case of COVID-19 infection was defined as a case with a positive result of viral nucleic acid testing in respiratory specimens. Serial interval (SI) was fitted by gamma distribution and considered the likelihood-based R0 using a branching process with Poisson likelihood. Seven days average of cases, deaths, doubling times and CFRs used to draw smooth charts. kernel density tool in Arc GIS (Esri) software has been employed to compute hot spot area of the study site.

**Results:**

The maximum-likelihood value of R0 was 2.88 (95%, CI: 2.57–3.23) in the early 14 days of epidemic. The case fatility rate for Alborz province (Iran) on March 10, was 8.33% (95%, CI:6.3–11), and by April 20, it had an increasing trend and reached 12.9% (95%,CI:11.5–14.4). The doubling time has been increasing from about two days and then reached about 97 days on April 20, 2020, which shows the slowdown in the spread rate of the disease. Also, from March 26 to April 2, 2020 the whole Geographical area of Karj city was almost affected by SARS-CoV-2.

**Conclusions:**

The R0 of COVID-19 in Alborz province was substantially high at the beginning of the epidemic, but with preventive measures and public education and GIS based monitoring of the cases,it has been reduced to 1.19 within two months. This reduction highpoints the attainment of preventive measures in place, however we must be ready for any second epidemic waves during the next months.

## Background

Coronavirus disease 201 (COVID19) is mainly a respiratory infection caused by SARS-CoV-2, an enveloped virus, containing a positive-sense single-stranded RNA. It could be transmitted by direct contact with the infected person’s respiratory droplets and contaminated surfaces. Fever, cough, sore throat, headache, fatigue, headache, myalgia, breathlessness and conjunctivitis are among the most common signs and symptoms (not in all) of COVID-19 patients [1–3].

The first confirmed cases of COVID-19 in Iran were reported on 19 February 2020 [4]. The number of COVID-19 daily new cases gradually increased until March 30 and reached 3186, and since then, the number of new cases has decreased. By 13^th^ April 275,427 RT-PCR tests were performed in Iran and a total number of 73,303 confirmed COVID-19 cases and 4585 related deaths were reported in Iran. In addition, 45,983 patients have been improved and discharged [4]. The first confirmed cases of COVID-19 in Iran were reported in Qom (a city is located 140 km to the south of Tehran). Subsequently, the neighboring provinces and gradually all 31 provinces of Iran were involved. Alborz province, which is 170 km north of Qom, was involved a few days after the first cases of the disease were reported in Qom. As regards to the number of confirmed cases and its related deaths, the Case Fatality Rate (CFR) of COVID-19 in Iran is estimated 6.2% by April 13 [5]. The global CFR of COVID-19 is estimated 6.3% by April 13 [6].

Epidemiologic data including indicators of epidemic spread speed and places can help us to know more about the disease and better control of the epidemic. The basic reproduction number (R0) is the number of secondary cases which one case would produce in a completely susceptible population [6]. The basic reproduction number of COVID-19 was estimated 2–2.5, indicating that 2–2.5 susceptible persons will be infected by an infected patient [7]. Given the rapid spread of the virus, the government immediately responded by establishing more than 60 laboratories to enhance the testing capacity, and consequently, there was a sudden spike in the reported number of positive cases.

Today, it is well proved that geographic information systems can provide epidemiologists and health professionals with a wide range of abilities to identify and solve health problems. Application of geographic information systems (GISs) in studies of infectious disease control results in a deeper approach to better planning for how to control them [8]. What distinguishes a geographic information system from other information systems is the existence of spatial analysis functions in that system, which are related to spatial location, and researchers are interested in analyzing their relationship with the incidence of diseases as well as the relationship between diseases and geographical features and find new ideas about the causes and patterns of disease [9].

The aim of this study was to investigate the epidemiology of COVID-19 and spatiotemporal distribution in Alborz province, as well as to estimate basic reproduction number (R0) and also modeling to estimate the incidence of the disease in future days.

## Methods

### Study sample and cases mapping

The study was conducted in all hospital of Alborz province, Karaj, Iran, which are designated to COVID-19 patients. Epidemiological data were extracted from electronic medical records of Alborz University of Medical Sciences, Karaj, Iran; using a standardized data collection form, All walk-in and referral patients who were diagnosed with COVID-19 according to WHO interim guidance, and those died between Feb 21, 2019 (when the first patients were diagnosed), and April 20, 2020, were included in our study. To ensure data quality and accuracy, some of the completed data forms were randomly selected, and their items were validated by an interviewer over the telephone. At the end of data collection, the forms’ data were entered into Excel and coded by two research assistants separately. In case of any discrepancy, a review of the forms, contacting the patients or their family, and/or exclusion of the participant were the next measures taken to resolve the discrepancies. The protocol of this study was reviewed and approved by the Institutional Review Board of Alborz University University of Medical Science (IR.ABZUMS.REC.1399.006).

The reported incidence cases of COVID-19 were mapped at the township level using Arc GIS software, from the beginning of the epidemic to the end of April 1, 2020. After cleaning and correcting errors, the data were entered into Excel data sheets using their home address or postal codes. The most recently updated electronic maps of Karaj city and its townships was used, and the map was linked to Excel with the join comment. A choropleth map, which uses a color range to show changes in the layers of polygons, was produced at the township level with a scale of 1/2000. COVID-19 maps are produced daily from the early stage of the disease. In the following, kernel density tool in Arc GIS (Esri) software has been employed to compute hot spot area of the study site. This tool calculates the density of points in a neighborhood around those points. For all maps, the hot spot areas are characterized by red.

### Case definition

A confirmed case of COVID-19 infection was defined as a case with a positive result of viral nucleic acid testing in respiratory specimens. Suspected case was defined as a case with symptoms of COVID-19 infection, but not confirmed by viral nucleic acid testing. All positive cases are systematically recorded in a designated registry which is used for follow-up and contact tracing. Serial interval (SI) was defined as the duration between symptom onset of the primary case and symptom onset of the secondary in a transmission chain.

### R0 and doubling time calculation

R0 was defined as the expected number of secondary cases that one primary case will generate in a susceptible population [10]. For testing, two respiratory tract samples (throat and nasopharyngeal swabs) are collected and submitted for viral nucleic acid testing. In this study, we used an informative prior distribution for the SI, which was estimated as 7.5 ± 3.4 days for COVID-19 in Wuhan, China [10], fit with a gamma distribution. We considered the likelihood-based R0 using a branching process with Poisson likelihood. Bootstrapping with 1000 times resampling was used for obtaining the distribution and confidence interval of R0. We then used the estimates of R0, SI, and daily incidence to simulate the trajectories and project the future daily cumulative incidence where the main assumption was that the model follows a Poisson distribution [11]. For each date 2, the number of incident cases I_t_ was drawn from a Poisson distribution with mean R_t_ ∑^t^_s=1_I_t-s_ W_s_, where R_t_ is the instantaneous reproduction number, W_s_ is the discrete SI distribution and I_t-s_ is the incidence at time step t – s.

For a 30-day projection, we used a uniform distribution of 0.8 to 1.5 for R0 and Bootstrapping with 1000 times resampling [12]. Doubling time is the time needed to double the cumulative number of patients. When doubling time increases means the epidemic is declining. Use of control measures and transmission rates can change doubling times of a disease across the time and between communities. Data on Alborz daily confirmed cases gathered from the surveillance system and used to calculate epidemic doubling times. World and Iran COVID-19 confirmed cases numbers gathered from WHO daily published data also used to calculate the doubling time as comparisons.

To determine how doubling time changes with time, calculations were done for each day since February 25, 2020 to April 16, 2020. The interval length (T1-T0) considered 5 days for each calculation.

The number of cumulative covid-19 patients in 2 perios of time (T1, N1 and T0, N0) used based on following formula to determine daily doubling times.

$${\text{Doubling Time}} = {\raise0.7ex\hbox{${{\text{T}}1 - {\text{T}}0}$} \!\mathord{\left/ {\vphantom {{{\text{T}}1 - {\text{T}}0} {\log_{2} \frac{{{\text{N}}1}}{{{\text{N}}0}}}}}\right.\kern-\nulldelimiterspace} \!\lower0.7ex\hbox{${\log_{2} \frac{{{\text{N}}1}}{{{\text{N}}0}}}$}}$$

Case fatality rates (CFRs) for Covid-19 were simply estimated for each day by dividing the accumulated COVID-19 confirmed deaths by accumulated confirmed cases. Although the method is not accurate, it is good enough for comparisons during the time and between different areas. Seven days average of cases, deaths, doubling times and CFRs used to draw smooth charts. Data analysis was performed using the “incidence”, “earlyR”, “ggplot2” and “projections” packages in R (3.6.3) software.

## Results

**Fig. 1:**
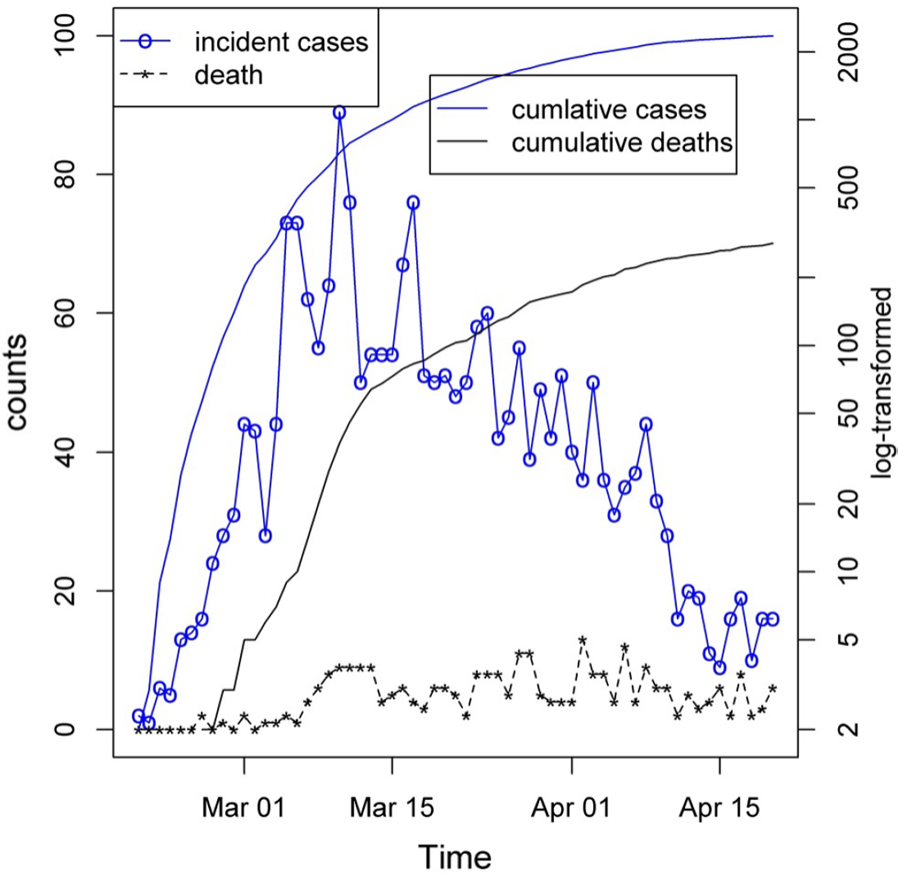
Frequency of daily mortality, confirmed positive cases, cumulative incidence of confirmed cases and related death from the beginning of the epidemic to April 20, 2020

This study showed that from the beginning of the outbreak until April 20, 2020, the total number of definitive positive cases and cases of severe acute respiratory infection (SARI) hospitalized in the Alborz province was 2359 and 4771 cases, respectively. Also, the number of cumulative deaths from definitive positive cases of COVID-19 was 284, and corresponding deaths due to severe acute respiratory infections were 542 accordingly. Daily number of new cases and deaths in addition to cumulative incident and death cases was illuserated in Fig. 1. Figure 1 shows positive cases as well as confirmed death cases from the beginning of the epidemic to April 20 in Alborz province. As can be seen, the results showed an increasing trend, and during the 10 to March 18, 2020, the highest daily report of cases is recorded and then reached the plateau status, followed by a descending trend. The first cases of death in Alborz province were reported on February 26 and then gradually increased. The highest number of daily deaths observed in the period from March 26 to April 5, and it was almost a fixed trend and then decreased.

**Fig. 2:**
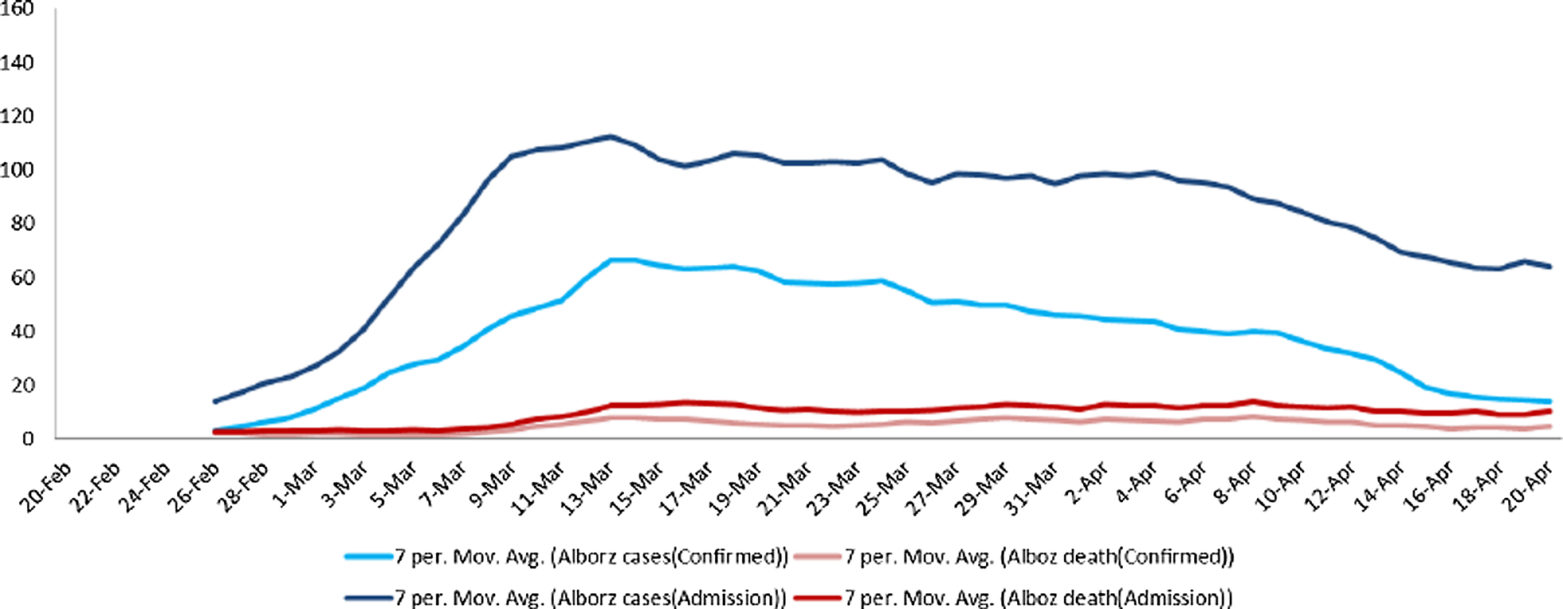
Case fatality rate of COVID-19 cases in the world, Iran and Alborz based on confirmed positive cases

Also, Fig. 1 shows the definitive cases of disease and cumulative death, which as of April 20 has reached 2199 definitive cases and 284 deaths from the disease. At the beginning of the outbreak, there were limitations in the performing and the accuracy of the molecular test, and patients with suspected respiratory symptoms were hospitalized based on the results of CT scans. Diagnostic molecular tests have been performed on all hospitalized individuals. Thus, the test results belong to a part of the patients who were hospitalized with a severe acute respiratory infection (SARI) and can’t generalize to the total patients in the whole province. Figure 2 shows the total daily confirmed cases and death in hospitalized patients in Alborz province. Dark blue lines indicate daily severe acute respiratory infections (SARI) and light blue lines reveal daily positive cases, and dark and bright red lines show daily deaths from severe acute respiratory infections (SARI) and death of positive cases, respectively. Although the observed pattern is different in terms of numbers, it shows almost the same trend in both cases.

The case fatality rate of COVID-19 was shown in Alborz province, Iran, and the world from March 10 to April 20 in Fig. 2. As can be seen, on March 10, Alborz province was 8.33% with a 95% confidence interval of (6.3–11), and by April 20, it had an increasing trend and reached 12.9% with a 95% confidence interval of (11.5–14.4). In Comparison of calculated death rate, the world and Iran has demonstrated that the reported rates for Alborz province have been higher; a same increasing trend was also detected in this period for world and Iran by 3.53% and 3.62% at the beginning of the interval to 6.8% and 6.23% on April 20 (Fig. 2).

### The trend of disease and projection of incidence cases based on confirmed positive and total cases (SARI)

#### A: Doubling time in Alborz province

**Fig. 3:**
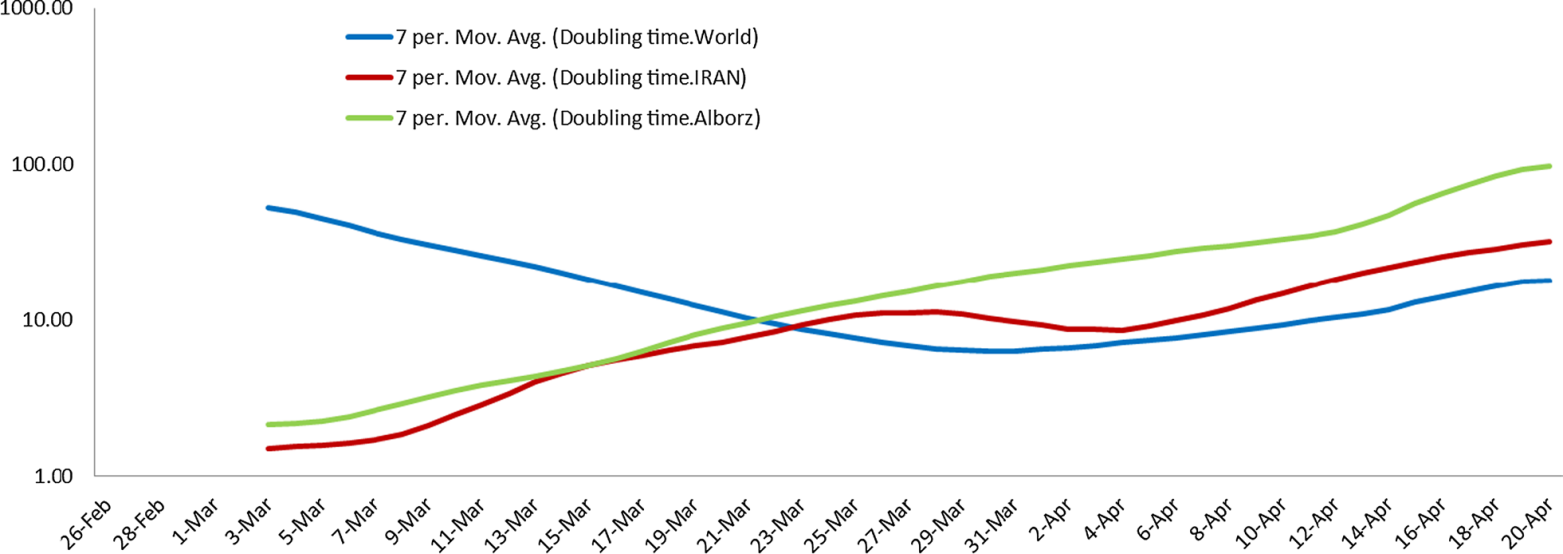
Doubling time of COVID-19 outbreak in the world, Iran, and Alborz (calculation with intervals of 5 days)

One of the indicators of disease spread is doubling Time. Figure 3 shows the COVID-19 trend in Alborz, Iran, and the world. The required time to double the number of patients in the world, as shown by the blue line, has been declining from 60 days on February 26 to about 6 days on March 24, suggesting an increase in the spread rate of the disease during this period and on April 20 it has reached 13 days (13.47 days) with a slow and increasing gradient. The red line indicates the status of Iran and shows the spread rate of the epidemic and the number of patients. The green line also shows the trend of Alborz province, which has been increasing from about two days in the range of February 26 and has reached about 97 days on April 20, which shows the slowdown in the spread rate of the disease.

#### B: Basic reproductive number (R0) and instantaneous reproduction number (Rt) In Alborz Province

**Fig. 4:**
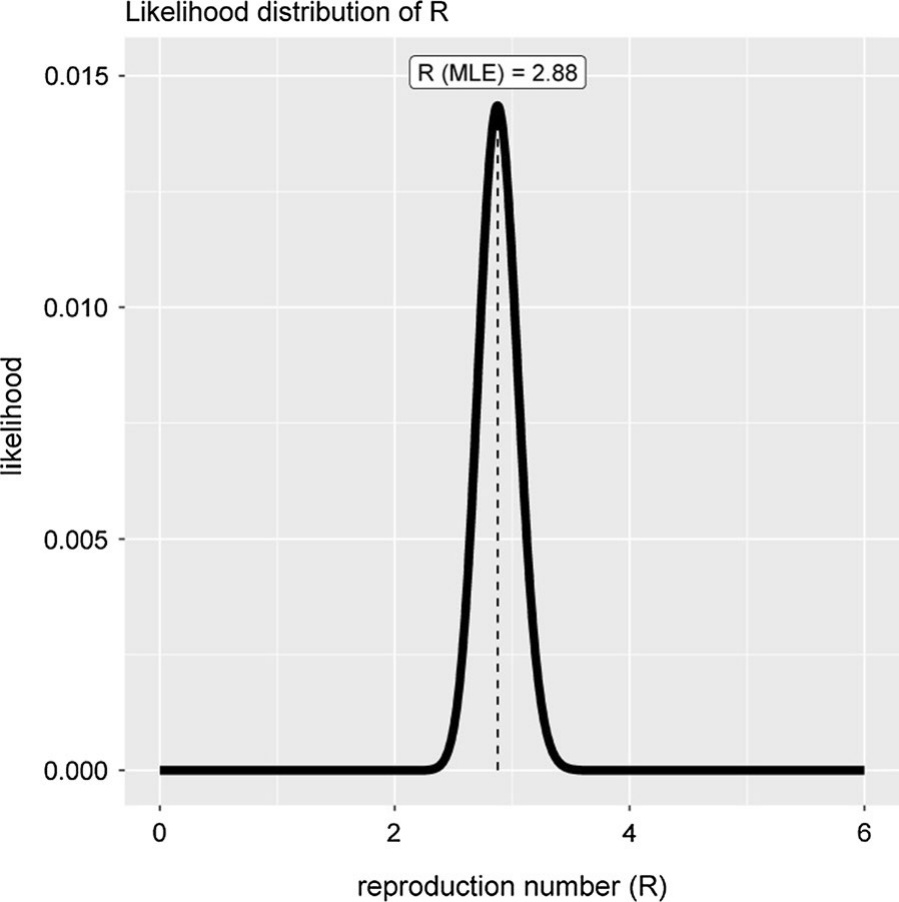
maximum likelihood estimation of basic reproductive number or the beginning of the epidemic (20 February and 02 March 2020)

**Fig. 5:**
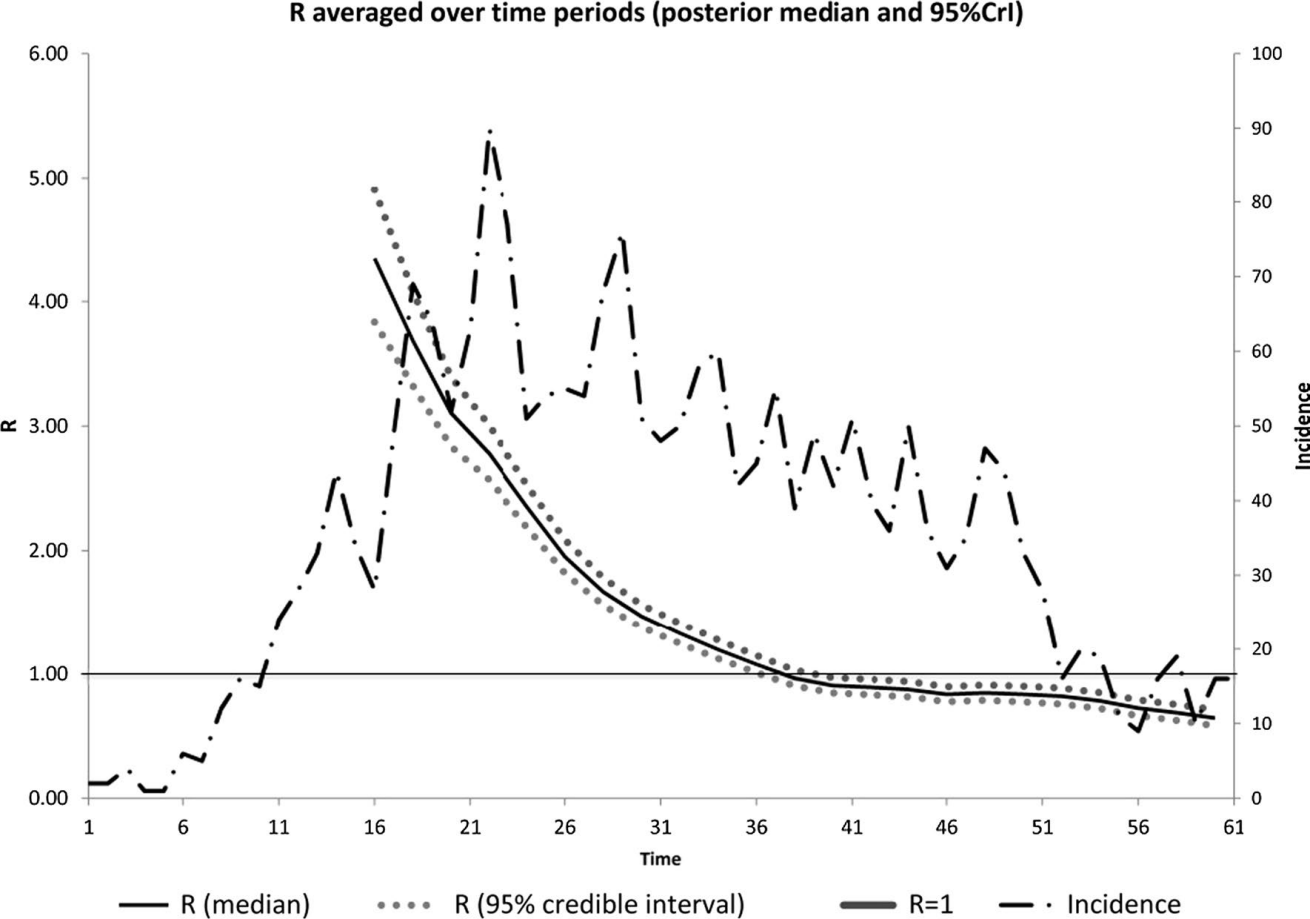
Instantaneous reproduction number (Rt) in Alborz Province, Iran

To estimate the basic reproductive number (R0), the gamma distribution, generation time equal to 7.5 (sd = 3.4) days was assumed. The maximum-likelihood value of R0 was estimated 2.88 (0.95 CI: 2.57–3.23) for 14 days (From 20 Feb, 2020 to 04 Mar 2020) after reporting the first cases in our population (Fig. 4). Calcution of Rt for this interval showed a deacrising patterns from day 36 of epidemic (Rt = 1.08) so that it was reached 0.65 (sd = 0.03) in day 61 of epidemic (Fig. 5).

#### C: Prediction of the number of new incidences for the next 30 days

**Fig. 6:**
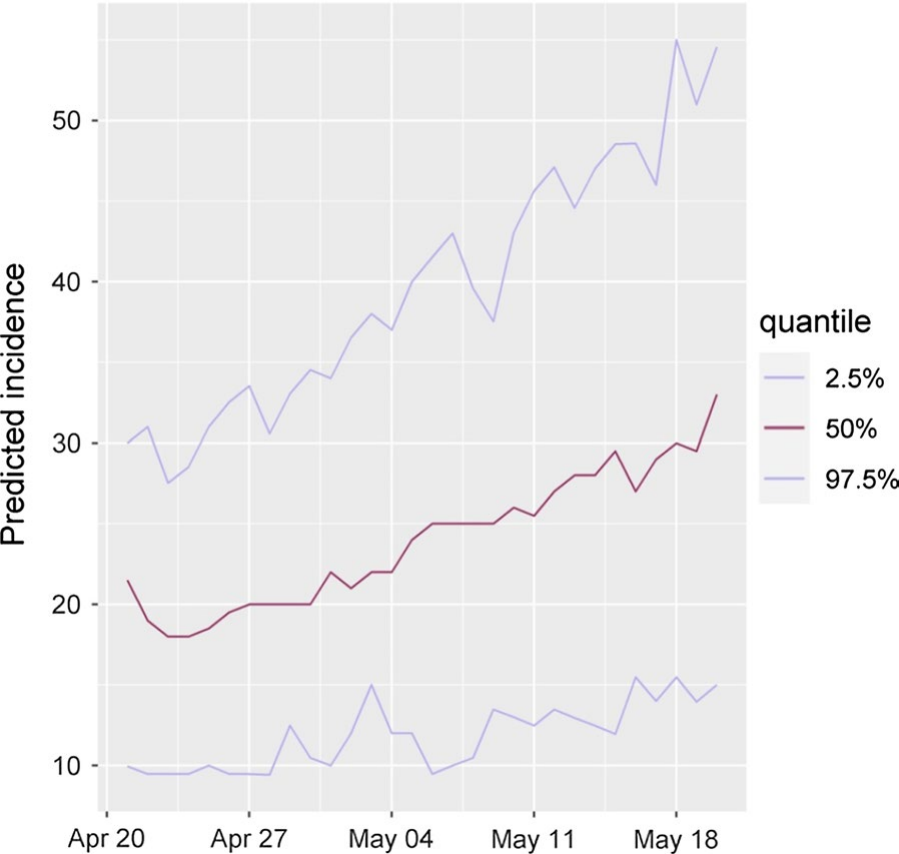
Predicting the number of daily new cases of COVID-19 in Alborz province until 20 May 2020

Prediction of daily and cumulative new cases have been undertaken in the next 30 days (by the end of 5 May 2020). Figure 6 shows the daily forecast for the number of new incidents from 21 April, 2020 to 20 May, 2020. A very crucial point in this prediction is the stability of people's hygiene behaviors and control measures of the competent authorities. In the next step, the cumulative rate at the end of the next month is calculated. The results show that if the current trend continues in this way, the mean daily incidence at the period will be 24.5 (sd = 8.7) cases.

As seen, the number of affected cases is estimated to be approximately 732, with a confidence interval of 493 to 993. If 20 percent of these cases require hospitalization, the necessary facilities for hospitalization and treatment of 146 people in the whole province should be considered. Based on the estimations and opinions of experts, it can be noted that at least about 15% of hospitalized people, i.e., about 22 people, need an intensive care unit bed that should be thought-out.

#### C: Infectiousness period

Given the calculated probability of virus infectivity, the highest risk of transmission is from the 7th day of infection to the next 14 days, with the interpretation that the highest probability of transmission is the tenth and eleventh days (nearly 100%) and the lowest probability of infection is reckoned to be for the next 20 days (less than 15%).

(Fig. 7).

**Fig. 7:**
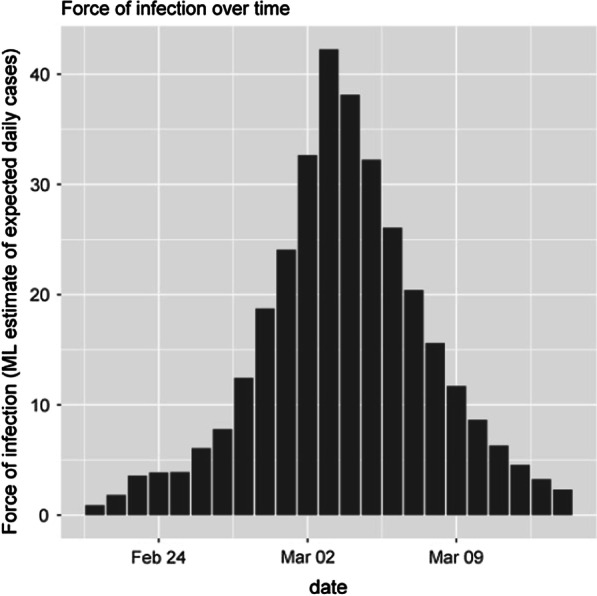
Infectiousness period of COVID-19 patients in Alborz province

#### Section 3: Spatiotemporal distribution of confirmed COVID-19 cases, geographic information system (GIS)

In this report, the location of each patient was mapped by the address of residence as well as their postal code on the Geographic Information System (GIS), and then daily maps of the distribution of the disease were prepared as vectors. Vector maps exclusively show the location of each patient. To analyze and compare the spread of the disease, it is necessary to produce raster maps. From the combination of vector layers of the network of passages, mosques, banks, hospitals, parks, bus stations, and terminals with disease distribution maps, the raster maps of the severity of the disease was prepared. Raster maps allow us to perform spatial analysis in the study area, observe how the disease has spread across the region, and identify high-risk areas.

**Fig. 8:**
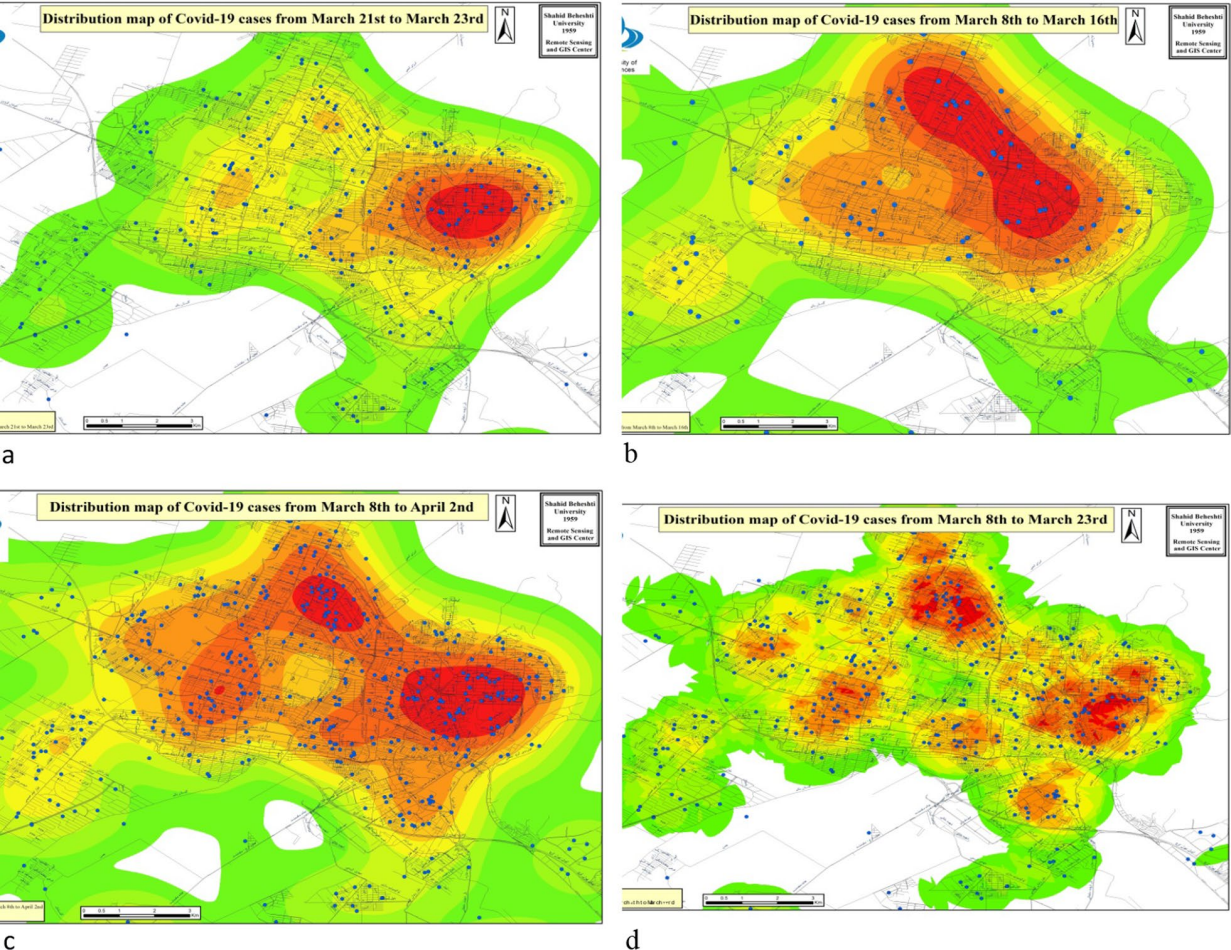
**a** Hot spot and distribution COVID-19 new cases of Karaj city March 21, 2020 to March 23, 2020; **b** Hot spot and distribution COVID-19 new cases of Karaj city from March 8 to March 16, 2020; **c** Hot spot and distribution COVID-19 new cases of Karaj until April 2, 2020; **d** Disease distribution and hot spot map in Karaj city from March 26 to April 2, 2020

Maps one to four demonstrate the rate of disease spread in the metropolis of Karaj from 8 March to 2 April 2020. The disease spread first to the north and west and then to the entire city. One of the important results of monitoring the high-risk areas of Karaj metropolis using the geographic information system is the use of information obtained from regular and daily monitoring of new cases and submitting its report to the university's health deputy to track contacts and disinfection of these patients (Fig. 8).

During this time, the more new case has been found in western Karaj and in the area of Shahid Givehchi Boulevard, Basij Boulevard, and Moffateh Street. Figure 8a. shows the disease severity map until March 23, 2020. Comparing this map with Fig. 8b signifies that in this 3-day gap, the disease also moved East and South of the city and areas such as Taleghani Street, Dr. Mofteh Street, and Esteghlal Boulevard in the East of the city and 45 Meters of Golshahr, Danesh Blvd., Haft e Tir Blvd. The south of the city is also polluted.

## Discussion

First Iranian COVID-19 cases were reported in Qom province on February 19, 49 days after identification of the first case in China [13]. Since then, SARS-CoV-2 has spread in Iran's provinces rapidly.

In this study, we evaluate COVID-19 early phase epidemic characteristics in Alborz province in central part of Iran. Alborz province, also called small Iran, has the highest number of immigrants from all over the country and is the home to the most ethnically diverse populations in Iran. Karaj is the capital and most-populous city of this province. Eastern region of the city is the oldest part, which has the highest population density and largest household size, while wastern region is new urban districts which financial district of Karaj is located. Overall, socioeconomic situation of the people living in northern rigions are higher compared to citizens living in southern parts [14, 15]. It has been proven that lower socioeconomic situation is associated with higher risk of COVID-19 infection and death.

First case of COVID-19 was diagnosed on Febuary 19 in iran. So as to control the pandemic several national and local control measures have been taken in Alborz province. initially on February 23rd local government decided to close all schools and universities. Then on March 7th travel bans started followed by restrictions and limitations in offices’ worktime since March 9th. Eventually national holidays and nationwide restrictions began on March 19th and continued until april 4th.

The prediction of the maximum number of infected patients, and more importantly, the maximum number of patients who will require intensive care is challenging. These predictions are crucial in planning facilities in Alborz province and the readiness of their hospitals. To predict trends of the epidemic, we conducted a GIS and estimated R0 and doubling time.

This study revealed R0 of COVID-19 infection in three consecutive periods within the first month in Alborz, 2.40, 1.70, 1.54, respectively. This estimation approximates WHO findings of the COVID-19 epidemic in china on January 18 (1.95 95%CI, 1.4–2.5) [16]. Liu et al. analyzed 12 publications that had been estimated R0, in the first month of the epidemic in China and demonstrated that R0 median and IQR are 2.79, and 1.16, respectively [17].

A recent study of 12 models of studies in Europe until April 7, 2020, reported the mean R0 value of COVID-19 about 3.28, with a median of 2.79 [18]. Moreover in Italy reported R0 values were in the range of 2.7 to 3.10 during the epidemic phase of the disease [19].

Although R0 may be a biological reality, this value is usually calculable with complicated mathematical models developed using numerous sets of assumptions. The interpretation of R0 estimates derived from totally different models needs associate understanding of the models’ structures, inputs, and interactions. as a result of several researchers using R0 haven't been trained in refined mathematical techniques, R0 is well subject to misrepresentation, misinterpretation, and misapplication. The variations in R0 values is also attributed to different ways and models of R0 estimation. R0 can be misrepresented, misinterpreted, and misused in a variety of ways that distort the metric’s true meaning and value. Because of these various sources of misperception, R0 must be applied and discussed with cautiousness in research and practice [20–23]. Regarding contagiousness disease, R0 is dependent on the rate of contact of people in the community, probability of transmition in each contact and duration of infectiousness, so applying approaches to reduce social contacts plays an important role in reducing this index [24]. The decrease in R0 value in this study upon time may be attributed to the enhancement of social distancing and self-quarantine policies. Specific conditions must be met for a valid estimation. These conditions are: a. complete detection of cases in the early days of the epidemic, b. calculation for a small timeline, and c. using an appropriate estimation method. R0 can be different according to the patterns of people’s contacts, structure of population and different subpopulations. In early stage of an epidemic, precisely estimating R0 is problematic, because the exact number of cases is not clear [25]. Yang et al. showed that by a self-quarantine, R0 value declines from 3.77 to 3.00 [26]. The physical distancing, quarantine, closing schools, and workplace distancing have been shown that were effective in decreasing R0 in Singapore [27]. As the number of people who became probably immune during the study is a small percentage of Alborz population, its contribution to R0 change seems negligible.

An increased doubling time of cases was observed within a month in the early stages of the epidemic in Alborz province.

In the early phase in China, the Doubling time was about two days [28], while, Chinazzi et al. showed that the travel ban in china increased the doubling time of the COVID-19 epidemic about 5 days [29]. Also, increasing of Doubling time by social distancing has been observed that suggests slowing down of epidemics of COVID-19 from January 20 to February 9, 2020, in China [30].

We can conclude from these findings that travel restrictions and social distancing increase the doubling time, causing an enhancement in healthcare systems' response to COVID-19 patients' requirements.

From March 10 to April 20, the mortality rate increased Alborz province from 8.33 to 12.9% that was higher than this index in the world and Iran in this period. This index has been ultimately affected by the number of sampled cases and considering that in the country and Alborz mostly, cases of hospitalized patients that are more severe are being tested, and the differences observed with the statistics of the whole world can be justified. However, in the case of a similar method and cases of testing, this index can show the quality of care, change in the behavior of the pathogen, or individual differences in response to the disease [31]. A study suggested that the exact mortality rate of COVID-19 has not been yet determined. Incomplete data and differences in testing standards could affect this rate [18].

One of the most critical issues in public and environmental health in the COVID-19 epidemic is planning, monitoring, and evaluation of health programs. Geographical information systems (GIS) allows rapid response and provide information about the epidemic dynamics to control the outbreak [32, 33]. GIS technology was used to assess the entire process of the SARS-CoV-2 outbreak. Our findings demonstrate north to west and west to the east path of the epidemic, concluding that the incidence of COVID-19 is higher in dense areas of Karaj city, especially in eastern Karaj, which most of the population in the city are situated. While it took about two and a half years for MERS and four months for SARS to infect 1000 people, the novel SARS-CoV-2 infected about two-million people worldwide in 4 months. While SARS-CoV-2 spreads rapidly, information has to move even faster. This is where map-based dashboards become crucial. In this study, we introduce GIS as an essential and useful tool in tracking and fighting against the SARS-CoV-2 outbreak [34, 35].

We predict that before May 30, 146, new COVID-19 patients will be referred to our hospitals daily. We suggest that the health care system in Alborz province should provide requirements for hospitalization of 728 new COVID-19, 109 of those need intensive care. This prediction is highly dependent on the authorities and population compliance with social-distancing policies [36, 37]. Prior studies showed that social distancing policies could avert cases by 20% and hospitalizations and deaths by 90% [38].

There are two things we must consider these estimations. First, in theory, we can estimate how much percent of the population needs to be immune to create herd immunity based on R0. In the real world, estimations are more complicated. Populations usually don’t mix randomly and covid19 infected people may have imperfect immunity.

Second, the trend of changes in R0 and doubling times, and also the estimation of patients load in the future, is related to the type of prevention measures and how much people commit to them during periods used to calculate the indicators. So, change in measures and people's behaviors may alter assumptions and predictions. It seems that with better access to experimentation and an increase in the number of cases examined in terms of coronavirus, the numbers are becoming more realistic now because not all patients are tested, calculations are affected by error and bias.

## Conclusion

In conclusion, the R0 of COVID-19 in Alborz province was substantially high at the beginning of the epidemic, but with preventive measures and public education and GIS based monitoring of the cases,it has been reduced to 1·19 within two months. This reduction highpoints the attainment of preventive measures in place, however we must be ready for any second epidemic picks during the next months. Mass screening of suspected cases, implementing travel restrictions especially during officially holidays, close contact tracing and expanding coronavirus testing to the community.

## Data Availability

The datasets generated and analyzed during the current study are available from the corresponding author on reasonable request.

## Abbreviations

R_0_: Basic reproduction number
GIS: Geographical information system
SI: Serial interval
R_t_: Instantaneous reproduction number
CFR: Case fatality rates
